# 

**Published:** 1908-08-15

**Authors:** 


CONTENTS.

PAGE

**The Mode of Entry of the Tubercle Bacillus** ... 511
**The Inheritance of Insanity** ... ... ... ... ... 512
**Annotations**—
The Mortality Amongst Medical Men—Vaccination in New Zealand—The Bishops' Appeal ... ... ... ... ... 313
**Medical Opinion and Movement** ... ... ... ... 514
**Hospital Clinics**—
"Let There Be Light." By J. F. Goodhart, LL.D., M.D., F.R.C.P. ... ... ... ... ... ... ... ... 517
**Medicine**—
Enlargements of the Spleen—IV. ... ... ... ... 521
Myocarditis and Myocardial Diseases—II. ... ... ... ... 522
**Surgery**—
Some Aspects of Intestinal Obstruction ... ... ... ... 523
**Laryngology and Rhinology**—
The Laryngeal Affections of Voice-Users ... ... ... ... 524

**Gynæcology**—
Pelvic Hæmatocele; with a Somewhat Unusual Case ... ... 525
**Ophthalmology**—
Cyclitis and Irido-Cyclitis ... ... ... ... ... ... ... 526
**Diseases of Children**—
The Value of Buttermilk in Disease ... ... ... ... ... 527
**Therapeutics**—
Prescriptions and Dispensing ... ... ... ... ... ... 528
**The General Practitioner's Column**—
A General Practitioner's Views about Locums ... ... ... 529
**New Appliances and Things Medical** ... ... ... 530
**Hospital Administration**—
Some Modern Continental Hospitals... ... ... ... ... 531
**News and Coming Events** ... ... ... ... ... 533
**Nursing Administration**—
Tradesmen's Books ... ... ... ... ... ... ... ... 535
**Reviews** ... ... ... ... ... ... ... ... ... 536

